# *Streptococcus agalactiae* meningoencephalitis associated with gastroesophageal reflux disease and chronic proton pump inhibitors use, in a 9 month-old infant: a case report

**DOI:** 10.1186/s12887-018-0995-0

**Published:** 2018-02-01

**Authors:** Victoria Bîrluţiu, Codruța Mihaela Luca, Rareș-Mircea Bîrluțiu

**Affiliations:** 1 Lucian Blaga University of Sibiu, Faculty of Medicine; Infectious Diseases Clinic, Academic Emergency Hospital Sibiu, Alba-Iulia Str. No.79 23/8, 550052 Sibiu, Romania; 2 Pediatric Infectious Diseases Clinic, Clinical Pediatric Hospital, Sibiu, Romania; 3 Lucian Blaga University of Sibiu, Faculty of Medicine; “FOISOR” Clinical Hospital of Orthopedics, Traumatology and Osteoarticular TB Bucharest, Sibiu, Romania

**Keywords:** *Streptococcus agalactiae*, Meningoencephalitis, Gastroesophageal reflux disease, Proton pump inhibitors, Infant

## Abstract

**Background:**

*Streptococcus agalactiae* (Group B *Streptococcus*) is recognized as the etiologic agent of newborn and infant meningitis, aged up to 90 days, starting from the colonization of the maternal genital or gastrointestinal tract, but it is rarely responsible for meningitis in old infants.

**Case presentation:**

We present the case of a 9 month-old infant diagnosed with *S. agalactiae* meningoencephalitis associated with chronic gastroesophageal reflux disease treated with a proton pump inhibitor (PPI).

**Conclusion:**

The use of a PPI is a risk factor for ultra-late onset of Group B *Streptococcus* meningitis. The use of PPI in infants should be closely monitored in the light of changes in the gut microbiota, in the oropharyngeal and of the respiratory tract colonization, potentially with pathogenic flora.

**Electronic supplementary material:**

The online version of this article (10.1186/s12887-018-0995-0) contains supplementary material, which is available to authorized users.

## Background

*Streptococcus agalactiae* (Group B *Streptococcus*, GBS) is a gram positive encapsulated coccus which has the ability to invade host tissues and the host defense mechanisms, to cause systemic infections such as sepsis, pneumonia, and due to its ability of crossing the blood brain barrier, it is responsible for meningitis, being the leader in the etiology of neonatal meningitis [1]. There are 10 serotypes identified, labeled Ia, Ib, II-IX [2], differentiated by the arrangement of four monosaccharides: glucose, N-acetylglucosamine, galactose and sialic acid, in the repetitive unit, the last monosaccharide being known to have an antiphagocytic action. Among the factors involved in the pathogenesis, it is generally accepted the important role of β-haemolysin/cytolysin toxin in producing brain endothelial cells [3], neuronal, astrocytal [4] and leptomeningeal lesions. GBS meningitis occurs as early-onset disease (EoD), within 7 days postpartum, by infection in utero, by placenta infection. It can be acquired perinatal during passage through the vaginal canal. Prematurity and low birth weight are additional risk factors. Late-onset disease (LoD) is associated with serotype III, and occurs after 7 days of life [5], up to 3 months, with 50% of infections being meningitis. GBS meningitis over 3 months (ultra-late onset disease -ULOD), represent about 6% of the cases. In Europe, serotype III is involved most often, in 70 to 81% [6], followed by serotype I in 13% of meningitis cases [7]. In the United States, most often are involved Ia, Ib, II, III and V serotypes in 96% of GBS meningitis cases [8]. Regarding older children up to the age of 18, there are few references that describe these cases though the numbers are small [9]. The host factors that should prevent the invasion of the GBS in the blood are neutrophils and macrophages, cells that ensure the bacterial clearance. Leukopenia in our case suggests a lack of defense by phagocytosis, which allowed the presence of bacteremia and subsequent the crossing of blood-brain barrier by GBS. The use of PPIs is recognized as a potential trigger of damages in the leukocytes functions, involving a decreased bactericidal activity [10], with an increased risk of enteric infections (3 times more) [11], including *Clostridium difficile* infections, the oropharyngeal colonization and the loss of bacterial diversity [12]. Prolonged use of PPIs is associated with pulmonary translocation of potentially pathogenic bacteria from the colonization of the gastric mucosa via the upper digestive and upper respiratory tracts and with the occurrence of community acquired pneumonia [13–16] namely with *Streptococcus pneumoniae* [17]. Prolonged administration of PPIs is also associated with hypomagnesaemia [18], iron deficiency [19], vitamin B12 deficiency [20, 21], osteoporosis [22, 23], atrophic gastritis, prolonged hypergastrinemia, and carcinoid formation. Gastroesophageal reflux disease associated with PPIs use, was in our opinion, the determinant factor of the upper digestive tract colonization with GBS, which was associated with leukopenia induced by PPIs, factor that is involved in bacterial clearance in the bacteremia stage.

## Case presentation

We present the case of a male Caucasian infant, aged 9 months and 3 weeks, who is brought to the emergency room having 40 °C fever, vomiting associated with breast feeding, restlessness, symptoms with an onset in the last 12 h, and associated with the mother’s referral as for the protuberant fontanelle. At the time of admission, the infant was febrile 38.8 °C, pale, with perioral cyanosis, with a normal lung assessment, relative bradycardia - heart rate (HR) of 88 beats per minute (bpm), with asymmetric deep tendon reflexes more diminished on the right side, right Babinski reflex present, right hemibody motor deficit, bulging of the anterior fontanelle, and Glasgow Coma Scale of 8. He was born naturally, normal birth weight, with food diversification started at the age of 6 months, and still breast fed at the moment. The only condition in the infant’s history is a gastroesophageal reflux disease (GERD), diagnosed at the age of 2 months, for which the patient received a treatment with esomeprazole 0.5 mg/kg/qd (5 mg/qd) for the last 4 months. The GERD improved after food diversification was introduced, but some inconstant vomiting is still present. Laboratory investigations revealed the following alterations: leukopenia – white blood cells 2.6 × 10^9^/L, 88.2% (2.293 × 10^9^/L) neutrophils, hemoglobin 9.9 g/L, hematocrit 28.8%, mean corpuscular volume 70.6 fL, platelets 133 × 10^9^/L, blood protein 5.74 g/dl, blood glucose 102 mg/dl, C-reactive protein 215 mg/L (references value 0–10 mg/L), and procalcitonin 10 ng/ml (references value < 0.5 ng/ml).

A chest radiography was performed and revealed an emphasized bilateral infrahilar and hilar interstitial drawing.

A cranial computerized tomograpohy (CT) scan was performed and revealed the following aspect: a diffuse cerebral edema with a wide left parietal hypodense area.

**Fig. 1:**
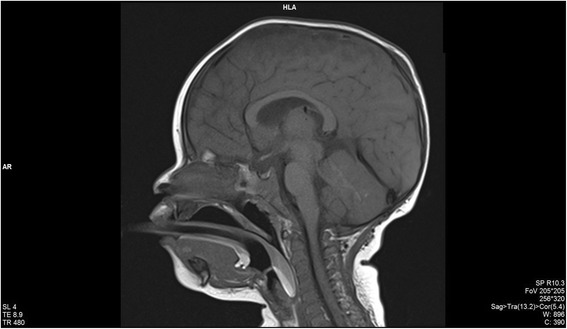
T1 MRI sequence, sagittal plane

**Fig. 2:**
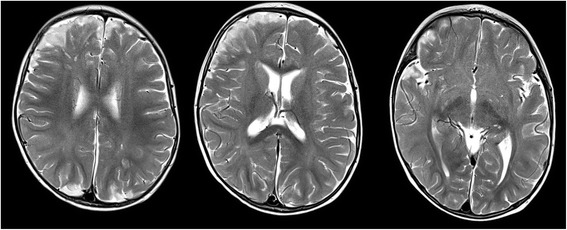
T2 MRI sequences, transverse plane

A cranial magnetic resonance imaging (MRI) was also performed and the findings were the following: a diffused swelling aspect of the cortical areas (hyposignal T1/T2 and hyperintensity on FLAIR sequences) with the disappearance of cortical sulci, and with the blurring of the cortical gray and white matter border in the left paramedian areas of the frontal, parietal, and occipital lobe. Hypersignal T2 and hyperintensity on FLAIR sequences in the fronto-parietal sulcus (bilateral) with a haematic component in magnetic susceptibility sequences. Ectasia of the small cortical cerebral veins, without cerebral lacuna images at the level of high flow cortical veins or venous sinuses. Small images with restricted diffusion spread across the bilateral supratentorial brain parenchyma. The administration of gadolinium-based contrast agent highlighted small diffused areas in the right frontal lobe white matter right, in the left frontal and parietal lobe, underlying areas of edema and at the level of the meninges, predominantly in the frontal and parietal lobes. See Figs. 1 and 2.

**Fig. 3:**
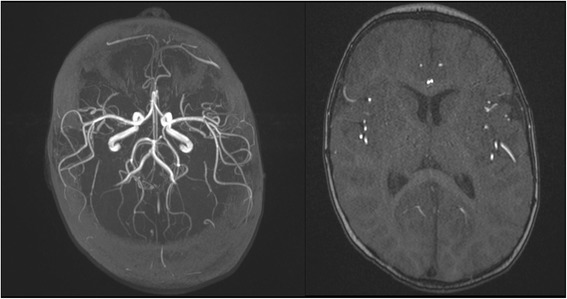
3D Time of flight angiography (TOF) MRI sequence, transverse plane

3D Time of flight angiography TOF sequences revealed no abnormalities in the cerebral arterial circulation. The conclusion of the MRI was: the described aspect corresponds to a meningoencephalitis. See Fig. 3.

A transfontanelar puncture was performed, and revealed a purulent cerebrospinal fluid (CSF), a CSF on examination with 310 WBCs/mm3 (reference values 0 to 10), a glucose of 5 mg/dL (reference values 40 to 70 mg/dL), a protein of 28.35 g/L (reference values 0.15 to 0.2 g/L), and with a latex agglutination test positive for group B *Streptococcus* (GBS). Gram stained smear shows a high number of leukocytes, rare red blood cells, and numerous Gram positive cocci arranged in pairs or short chains that were confirmed on growth medium as *Streptococcus agalactiae.* The antibiogram of the bacterial isolates determined by disc diffusion method revealed the sensitivity to ampicillin, linezolid, vancomycin, teicoplanin, trimethoprim/sulfamethoxazole, ciprofloxacin, and the resistance to tetracycline and clindamycin. The treatment was initialized with cerebral depletion therapy (such as administration of intravenous solutions like mannitol), ampicillin 200 mg/kg/day, ceftriaxone 90 mg/kg/day, and vancomycin 60 mg/kg/day. After receiving the susceptibility tests result for the isolated strain, ampicillin, and vancomycin were administered. Also, dexamethasone, and antiseizure drugs were associated to the treatment. The bacteriological examination of mother’s genital secretions, breast milk, and peri-areolar skin were negative. The bacteriological examination of infant’s gastric secretion, was unfortunately performed after initiation of the treatment and was negative. His response to the therapy was slowly favorable, the survival being associated with motor (right hemiplegia), language deficits, and episodic irritability (Additional file 1: Flow diagram).

## Discussion and Conclusions

The use of PPI in infants should be closely monitored in the light of changes in the gut microbiota, in the oropharyngeal and of the respiratory tract colonization, potentially with pathogenic flora. The use of this class of drugs for over a month would require a close monitoring of white blood cells counts, bacteriological examinations (throat swab, nasal swab, and so on) in order to detect a change in the bacterial spectrum with the predominance of one species with recognized virulence factors. It is more important to recognize that acid suppressing medications cause immune dysfunction that persists after discontinuation of the drug, therefore, physicians should be judicious in prescribing these medications.

## Additional file

Additional file 1: Flow diagram. (DOCX 44 kb)
