# Supplementary material for: Streptococcus agalactiae meningoencephalitis associated with gastroesophageal reflux disease and chronic proton pump inhibitors use, in a 9 month-old infant: a case report
Source: BMC Pediatr. 2018 Feb 1;18:21. doi: 10.1186/s12887-018-0995-0 (PMC5796439; doi:10.1186/s12887-018-0995-0)
Supplement: Additional file 1: — Flow diagram. (DOCX 44 kb) [file 12887_2018_995_MOESM1_ESM.docx]

**Treatment is initiated**

**On the 3rd day of admission a MRI is performed**

**Treatment is continued**

**GCS 8 , seizures**

**Antiseisure drugs are added**

**On the 1st day of admission:**

**Other blood works are performed**

**A cranial CT scan is performed**

**A transfontanelar puncture is performed**

**Antibiotic treatment is started**

**Admission into the hospital**

**oct 2015:**

**40°C fever, vomiting associated with breast feeding, restlessness, symptoms with an onset in the last 12 hours, and associated with the mother's referral as for the protuberant fontanelle**

**In ER blood tests are performed**

**Age of 5 months:**

**treatment with esomeprazole 5 mg/day is initiated**

**Age of 2 months:**

**Diagnosed with gastroesophageal reflux disease**

**BIRTH**

**DEC 2014**

**born naturally, eutrophic, with food diversification started at the age of 6 months**

**On the 6th day of admission**

**GCS 12**

**Foal seizures**

**Biological status of the patient is closely monitored**

**Therapy is closely monitored and adjusted according to the clinical evolution and to the performed testes and there results**

**On the 7th day of admission:**

**No seizures**

**Normal fontanelle**

**On the 9 day of admission a cranial CT scan is performed**

**On the 11 day of admission:**

**conscious, active motility - reduced at the lower left lower limb associating hyperreflexia, right eye strabismus**

**Medical recovery treatment is initiated**

**On the 16 day of admission**

**Active mobility of the superior limp is recovered with a small deficit at the lower right limb**

**After 28 day of admission the patient is discharged from the hospital.**

**Biological status within normal limits.**

**He continues the medical recovery procedures as an outpatient**

**Writing case report**

**TIME**

| CRP  mg/L | WBC  X 10^9^/L | NEUTROPHILS  X 10^9^/L | HGB  g/L | PLT  X 10^9^/L | PCT  ng/mL | FIBRIN MONOMERS |
| --- | --- | --- | --- | --- | --- | --- |
| 215 | **2.6** | **2.28** | **9.9** | **133** | **10** | **-** |
| 215 | **6.39** | **4.0** | **-** | **-** | **10** | **-** |
| - | **21** | **11.8** | **8.8** | **107** | **-** | **++++** |
| - | **11.7** | **6.9** | **9** | **453** | **-** | **-/++++** |
| 19 | **14.5** | **3.6** | **8.4** | **131** | **0** | **-/++++** |

An overview of some biological parameters of the patient.
